# Radical pericardiectomy for the treatment of constrictive pericarditis: Single-center experience in Vietnam

**DOI:** 10.21542/gcsp.2024.45

**Published:** 2024-11-01

**Authors:** Phan Quang Thuan, Tran Quoc Han, Ho Duc Thang, Nguyen Hoang Dinh

**Affiliations:** 1 Department of Adult Cardiovascular Surgery, University Medical Center, Ho Chi Minh City, Viet Nam; 2 Department of Cardiovascular and Thoracic Surgery, Faculty of Medicine, University of Medicine and Pharmacy at Ho Chi Minh City, Viet Nam

## Abstract

Background: Constrictive pericarditis (CP) is a chronic inflammatory condition often necessitating surgical intervention. Radical pericardiectomy is the standard treatment, but the use of cardiopulmonary bypass (CPB) varies based on intraoperative hemodynamics. This study aims to evaluate the effectiveness of radical pericardiectomy combined with CPB and the apical suction device in treating CP.

Methods: We conducted a single-center retrospective analysis of 10 patients undergoing radical pericardiectomy for CP. Clinical data, surgical details, and postoperative outcomes were collected. Follow-up assessments included echocardiographic and clinical evaluations at 3 months, with a mean survival follow-up duration of 30.9 ± 21.3 months.

Results: Among the included patients, 60% underwent CPB during surgery. Despite longer recovery times and hospital stays, CPB usage did not increase postoperative complications. Echocardiographic and clinical assessments at 3-month follow-up revealed significant improvements in cardiac function and symptom relief. No cases of CP recurrence were observed during the follow-up period.

Conclusion: Radical pericardiectomy combined with CPB and the apical suction device demonstrates effectiveness in treating CP, with favorable short-term outcomes and low recurrence rates. Further studies with larger sample sizes and longer follow-up durations are warranted to validate these findings.

## Introduction

Constrictive pericarditis (CP) denotes a chronic inflammatory process culminating in progressive pericardial fibrosis, which encases the heart within a thickened and fibrotic pericardium. Consequently, there ensues impaired diastolic filling of the cardiac chambers, alongside elevated right atrial mean pressure and end-diastolic pressure in both ventricles, ultimately leading to diminished cardiac output.

According to the European Society of Cardiology (ESC) 2015 recommendations, radical pericardiectomy, which includes epicardium removal (class I, level C), is advised for chronic permanent CP^1^. In the world, the first authors Rehn and Sauerbruch in 1913 documented a successful pericardial resection for chronic constrictive pericarditis *via* a left anterolateral thoracotomy approach. Current studies advocate for midline radical pericardiectomy to attain superior long-term outcomes. To prevent bleeding, the European Society of Cardiology(ESC) recommends employing cardiopulmonary bypass (CPB) solely in cases accompanied by concurrent cardiac surgical lesions. However, a recent study has shown that utilizing CPB enables more extensive pericardial removal, thereby reducing the long-term recurrence risk without increasing the likelihood of bleeding^1,2^.

Herein, we present the outcomes of a case series involving radical pericardiectomy for constrictive pericarditis and consolidate the findings from a single center in Vietnam. Additionally, we describe the cases with or without the utilization of CPB support.

## Materials and Methods

### Patient selection and follow up

All patients diagnosed with CP who underwent pericardiectomy at our institution between March 2019 and May 2024 were included in the study. During this period, 10 patients were identified, and their data were collected retrospectively from case notes. Postoperative clinical and transthoracic echocardiographic (TTE) parameters were recorded. All patients were reviewed at 3 months postoperatively, with their clinical status, TTE results, and any complications documented. The surviving patients were followed for a mean duration of 30.9 ± 21.3 months.

### Surgical technique

**Figure 1.**
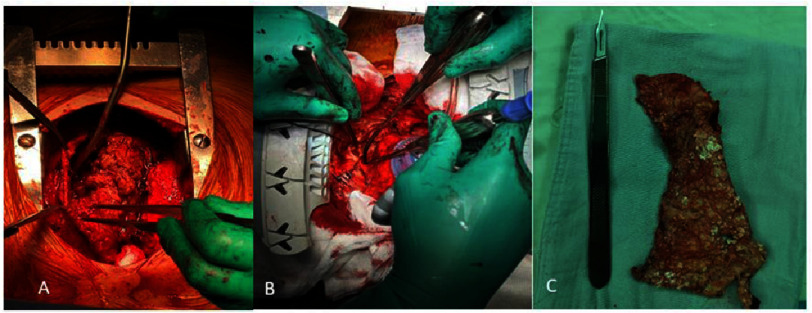
Intraoperative Imaging: A: Radical pericardiectomy, B: Suction device in apical position, C: Inflammatory pericardium.

After median sternotomy, radical pericardiectomy, inclusive of epicardium removal, is employed. In instances where altering the heart’s position does not impact hemodynamics, an apical suction device is utilized to elevate the heart position, facilitating access to the diaphragm and the left side. In challenging scenarios, cardiopulmonary bypass is implemented (Figure 1).

### Statistical analysis

Continuous variables were reported as either the mean ±standard deviation (SD) for normally distributed data or the median [interquartile range (IQR)] for non-normally distributed data. Categorical data were presented as proportions. Statistical significance was established at a *P*-value of <0.05. Calculations were performed using the R statistical software package.

## Results

### Demographic data

A total of 10 patients underwent radical pericardiectomy, with an average age of 38.6 ± 13.4 years and a male predominance of 60%. All patients presented with symptoms, with 60% categorized as NYHA class III and 40% as NYHA class II. Ascites was observed in 70% of patients, while pedal edema and hepatomegaly were present in 60% of patients each. Among the patients, 70% had cirrhosis, with 4 cases classified as Child-Pugh A and 3 cases as Child-Pugh B. Idiopathic etiology was the most common cause of constrictive pericarditis, accounting for 50% of cases, followed by prior cardiac surgery. Tuberculosis and recurrent pericarditis were also identified as potential causes (Table 1).

**Table 1:** Preoperative patient variables (*N*= 10).

	All cases (*N*= 10)	CPB (*N*= 6)	Non_CPB (*N*= 4)
Age: mean ± SD/median (IQR) (years)	38.6 ± 13.4	34 (29.7–42.5)	40.3 ± 17.7
Male	6 (60)	3 (50)	3 (50)
Body Max Index: mean ± SD (Kg/m^2^)	24.5 ± 5.1	22.2 ± 4.4	27.8 ± 4.7
**NYHA class**			
I	0	0	0
II	4 (40)	3 (50)	1 (25)
III	6 (60)	3 (50)	3 (75)
IV	0	0	0
**Aetiology**			
Tuberculosis (%)	1 (10)	1 (16.7)	0
Recurrent pericarditis	1 (10)	0	1 (25)
Prior cardiac surgery	2 (20)	1 (16.7)	1 (25)
Idiopathic	5 (50)	3 (50)	2 (50)
Pericardial mesothelioma	1 (10)	1 (16.6)	0
**Main symptoms**			
Dyspnea	10 (100)	6 (100)	4 (100)
Pedal oedema	6 (60)	3 (50)	3 (75)
Ascites	7 (70)	4 (66.7)	3 (75)
Hepatomegaly	6 (60)	4 (66.7)	2 (50)
**Cirrhosis** Child-Pugh A	4 (40)	2 (33.3)	2 (50)
**Cirrhosis** Child-Pugh B	3 (30)	3 (50)	0
Jugular venous distension	7 (70)	4 (66.7)	3 (75)
**Electrocardiogram**			
Atrial Fibrillation	2 (20)	1 (16.7)	1 (25)
Atrioventricular block	1 (10)	1 (16.7)	0

All patients underwent full sternotomy and radical pericardiectomy as the surgical technique. Sixty percent of patients underwent surgery with the assistance of cardiopulmonary bypass (CPB), while 40% utilized an apical suction device to facilitate changes in heart position. The average cardiopulmonary bypass time for patients undergoing CPB was 120 ± 33.6 min. The average operation time was 254 ± 55.6 min, with longer durations observed in the CPB group. The pathology of the patients predominantly consists of chronic pericarditis, with one case each of malignant mesothelioma and giant cell myocarditis, as well as one case each of acute pericarditis (Table 2).

**Table 2:** Postoperative outcomes (*N*= 10).

	All cases (*N*= 10)	CPB (*N*= 6)	Non_CPB (*N*= 4)
**Surgical**			
Apical suction device (%)	4 (40)	0	4 (100)
Time operation: mean ± SD (mins)	254 ± 55.6	276 ± 50.5	223 ± 52.6
Cardiopulmonary Bypass (CPB) time: mean ± SD (mins)		120 ± 33.6	
**Cardiovascular Surgery Intensive Care Unit (CSICU)**			
Ventilation Time: mean ± SD/median (IQR) (hours)	11 (5.5–18)	12 (5.5–50.2)	9.3 ± 4.3
Time CSICU (hours)	98.6 ± 70.1	128.5 ± 75.1	53.8 ± 29.4
Postoperative hospital stays (days)	26.1 ± 11.5	27 ± 13.6	24.7 ± 9.3
**Postoperative complications**			
Pulmonary complications (%)	1 (10)	1 (16.6)	0
(%)	2 (20)	1 (16.6)	1 (25)
Neurology accident (%)	1 (10)	1 (16.6)	0
Postoperative Bleeding (%)	1 (10)	1 (16.6)	0
Mortality (%)	0	0	0
**Pathology**			
Giant cell myocarditis (%)	1 (10)	0	1 (25)
Malignant mesothelioma (%)	1 (10)	1 (16.6)	0
Acute Pericarditis	1 (10)	0	1 (25)
Chronic Pericarditis (%)	7 (70)	5 (83.4)	2 (50)

### Perioperative outcomes

There were no cases of in-hospital mortality. Postoperative bleeding occurred in 10% of cases; however, none required reoperation. Ventilation time, time spent in the Cardiac Surgery Intensive Care Unit (CSICU), and postoperative hospital stays were all longer in the CPB group. One case experienced traumatic pneumonia, but the patient stabilized after prolonged mechanical ventilation. Twenty percent of patients developed renal failure but did not require dialysis. Additionally, 10% of patients experienced neurological complications, but without any sequelae (Table 2).

**Figure 2.**
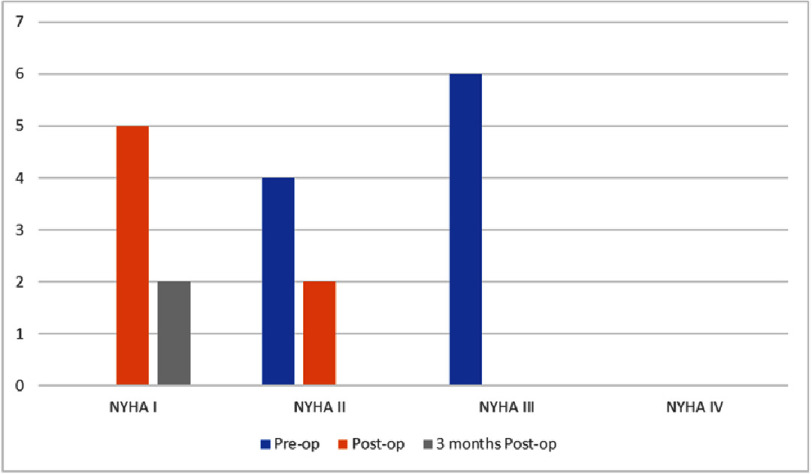
Symptomatic improvement after pericardiectomy for constrictive pericarditis, NYHA: New York Heart Association functional class (*N*= 10).

The left ventricular ejection fraction and fractional area change improved postoperatively and remained stable during the 3-month follow-up. The left ventricular end diastolic dimension improved immediately after surgery and maintained stability during follow-up. There was also an improvement in liver function after surgery and at the 3-month follow-up (Table 3). After surgery, patients’ symptoms improved to NYHA class I or II. Three months postoperatively, symptoms further improved, with only 20% of patients experiencing NYHA class I symptoms (Figure 2).

**Table 3:** Changes in echocardiography and laboratory monitoring parameters (*N*= 10).

	Preoperative characteristics	Preoperative characteristics	3 months Postoperative characteristics
**Transthoracic echocardiogram**			
Left Ventricular Ejection Fraction (LVEF %)	54.6 ± 10.5	60.7 ± 7.6	61.7 ± 7.9
Fractional Area Change (FAC %)	30.2 ± 12.1	42.8 ± 4.8	45.1 ± 4.9
Left Ventricular End Diastolic Dimension (LVEDD mm)	43.9 ± 5.1	48.3 ± 7.1	46.7 ± 6.7
**Laboratory**			
Albumin (g/dl)	34.3 ± 7.2	34.9 ± 4.3	44.8 ± 5.5
SGPT (U/l)	52.5 ± 41.6	42.9 ± 46.2	25.7 ± 7.4
Total Bilirubin (mg/dl)	3.6 ± 2.2	1.6 ± 1.1	1.1 ± 1.3

### Survival outcomes

After the follow-up period, there were no cases of death or recurrence of CP.

## Discussion

The average age of our patients was 38.6 ± 13.4 years, with the CPB group having a higher average age compared to the non-CPB group. Compared to the study by Nicola Vistarini et al., our patients were younger and had a lower proportion of men. This indicates that our patient population is more evenly distributed between men and women and consists of younger individuals, suggesting specific causes prevalent in developing countries. Nearly all studies by other authors report that patients exhibit symptoms of dyspnea ranging from New York Heart Association(NYHA) Classification I–IV^3,4^. In contrast, 100% of our patients experienced dyspnea, classified as NYHA II or III, and were often accompanied by symptoms of right heart failure such as pedal edema, ascites, and hepatomegaly. This indicates that patients in our country tend to seek medical attention only when their symptoms become intolerable.

Based on pathology results, idiopathic causes remain the primary cause of constrictive pericarditis at our hospital, consistent with global research findings. However, infectious causes are still significant contributors at our institution. In comparison to other studies, the infectious etiology observed in our cohort aligns with findings from Western authors. However, the limited sample size in our study precludes definitive conclusions, making it difficult to generalize these findings to the broader population in our country^3,4^. Additionally, malignant and cardiac surgical causes have emerged, similar to trends in developed countries^5,6^. Cardiac arrhythmias were observed in 30% of our patients, with a higher incidence in the CPB group. This indicates that preoperative arrhythmias contribute to hemodynamic instability during surgery, necessitating the use of CPB to facilitate radical pericardiectomy.

The use of CPB in radical pericardiectomy aims to achieve complete pericardiectomy in cases of hemodynamic instability. Although there is an increased risk of bleeding complications and mortality, current studies show that using CPB can lead to successful outcomes by minimizing the risk of recurrent constrictions and reducing the need for reinterventions^2,4^. Furthermore, bleeding complications are not significantly different from those in groups that do not use CPB^2^. For hemodynamically stable cases, the use of an apical suction device helps achieve radical pericardiectomy^7^. During surgery, if the patient becomes hemodynamically unstable when changing the heart’s position, we use CPB. If the hemodynamics are stable, we use the apical suction device (commonly used in CABG) to remove the pericardium below and to the left side of the heart, facilitating the radical pericardiectomy technique.

Our findings indicate that 60% of patients underwent CPB during surgery, while the remaining group utilized the apical suction device. At our center, apical suction is employed to optimize pericardectomy, particularly for achieving full excision of the pericardium along the diaphragmatic and left ventricular surfaces. In patients with stable hemodynamics who do not require cardiopulmonary bypass (CPB), apical suction is utilized intraoperatively to facilitate tissue manipulation and improve surgical efficiency. The CPB group experienced longer mechanical ventilation times, CSICU stays, and overall hospital stays. However, postoperative complications, particularly bleeding, did not significantly differ from the non-CPB group. This observation is consistent with studies conducted by other researchers globally^4,8,9^. Additionally, no cases of CP recurrence were recorded during the follow-up period, providing strong evidence for the efficacy of the radical pericardiectomy technique. Proper utilization of CPB is crucial, especially in patients with unstable hemodynamics during surgery, as these individuals typically present with severe clinical symptoms and arrhythmias prior to surgery.

Echocardiographic monitoring revealed significant improvement in both left and right heart function post-operatively and at the 3-month mark. Additionally, liver function test parameters exhibited notable improvement. Based on the NYHA classification, most patients experienced symptom relief, with only 20% remaining in NYHA class I at the 3-month follow-up. These findings align with research conducted by various authors worldwide. Importantly, no deaths or CP recurrences occurred during the follow-up period. While our results appear more positive compared to other studies, it’s essential to acknowledge the limitations: a small study sample and a relatively short follow-up duration. To establish the effectiveness of our CP treatment approach, further investigation with a larger sample size and longer follow-up is warranted.

## Limitations of this study

This study has several limitations. The small sample size reduces statistical power and limits generalizability. Being a retrospective single-center study, there is potential for selection bias, which may affect the applicability of the findings to other settings. The short follow-up period of three months also limits insight into long-term outcomes. Additionally, the small sample size made it difficult to apply more advanced statistical methods, such as non-parametric tests. Larger, multi-center studies with longer follow-up are needed to confirm these findings.

## Conclusion

Radical pericardiectomy remains the standard surgical approach for treating CP. The decision to utilize CPB during surgery depends on the patient’s hemodynamic status at the time of the procedure, with the ultimate goal being the complete removal of the pericardium. Although patients undergoing CPB experience longer recovery times and extended hospital stays, this technique does not significantly increase postoperative complications and effectively prevents CP recurrence. Our hospital’s experience with radical pericardiectomy demonstrates its efficacy when combined with both CPB and the apical suction device. However, to validate these results, a larger sample size and a longer follow-up period are necessary.

## Ethical Approval

The Ethics Committee of University Medical Center HCMC has reviewed this study.

## Competing interests

The author(s) declare no conflict of interest in preparing this article.

## Authors’ contributions

Data collection and writing: PQT and TQH. Critical review and revision: PQT and NHD. Final approval of the article: All authors. Accountability for all aspects of the work: All authors.

## Funding

No funding was received for this study.

## Availability of data and materials

All of the material is available and owned by the authors and/or no permissions are required.
